# Chemotherapeutic agents attenuate CXCL12-mediated migration of colon cancer cells by selecting for CXCR4-negative cells and increasing peptidase CD26

**DOI:** 10.1186/s12885-015-1702-2

**Published:** 2015-11-10

**Authors:** Murray J. Cutler, Erica L. Lowthers, Cynthia L. Richard, Dagmar M. Hajducek, Paul A. Spagnuolo, Jonathan Blay

**Affiliations:** 1 School of Pharmacy, University of Waterloo, 200 University Ave. W., Waterloo, ON N2L 3G1 Canada; 2 Department of Pharmacology, Dalhousie University, Halifax, Nova Scotia Canada; 3 Department of Statistics and Actuarial Science, University of Waterloo, Waterloo, Ontario Canada; 4 Department of Pathology, Dalhousie University, Halifax, Nova Scotia Canada; 5 Sim & McBurney/Sim Lowman Ashton & McKay LLP, Toronto, Ontario Canada

**Keywords:** Colorectal cancer, CXCL12, CXCR4, CD26, CD44, Cell migration, Metastasis

## Abstract

**Background:**

Recurrence of colorectal cancer (CRC) may arise due to the persistence of drug-resistant and cancer-initiating cells that survive exposure to chemotherapy. Proteins responsible for this recurrence include the chemokine receptor CXCR4, which is known to enable CRC metastasis, as well as the cancer-initiating cell marker and peptidase CD26, which terminates activity of its chemokine CXCL12.

**Methods:**

We evaluated the expression and function of CXCR4 and CD26 in colon cancer cell lines and xenografts following treatment with common chemotherapies using radioligand binding, flow cytometry, immunofluorescence, and enzymatic assays.

**Results:**

5-Fluorouracil, oxaliplatin and SN-38 (the active metabolite of irinotecan), as well as cisplatin, methotrexate and vinblastine, each caused decreases in cell-surface CXCR4 and concomitant increases in CD26 on HT-29, T84, HRT-18, SW480 and SW620 CRC cell lines. Flow cytometry indicated that the decline in CXCR4 was associated with a significant loss of CXCR4+/CD26- cells. Elevations in CD26 were paralleled by increases in both the intrinsic dipeptidyl peptidase activity of CD26 as well as its capacity to bind extracellular adenosine deaminase. Orthotopic HT-29 xenografts treated with standard CRC chemotherapeutics 5-fluorouracil, irinotecan, or oxaliplatin showed dramatic increases in CD26 compared to untreated tumors. Consistent with the loss of CXCR4 and gain in CD26, migratory responses to exogenous CXCL12 were eliminated in cells pretreated with cytotoxic agents, although cells retained basal motility. Analysis of cancer-initiating cell CD44 and CD133 subsets revealed drug-dependent responses of CD26/CD44/CD133 populations, suggesting that the benefits of combining standard chemotherapies 5-fluoruracil and oxaliplatin may be derived from their complementary elimination of cell populations.

**Conclusion:**

Our results indicate that conventional anticancer agents may act to inhibit chemokine-mediated migration through eradication of CXCR4+ cells and attenuation of chemokine gradients through elevation of CD26 activity.

**Electronic supplementary material:**

The online version of this article (doi:10.1186/s12885-015-1702-2) contains supplementary material, which is available to authorized users.

## Background

Current standard of care for late stage and metastatic colorectal cancer (CRC) includes surgical resection followed by adjuvant 5-fluorouracil (5-FU) plus leucovorin, and oxaliplatin (Ox) or irinotecan (IT) chemotherapy until progression [1]. However, the development of drug resistance and relapse with refractory disease limits the 5-year survival for metastatic CRC to less than 10 % [2].

Progression is driven in-part by the G-protein-coupled chemokine receptor CXCR4, with high expression of CXCR4 in CRC being associated with a greater risk of recurrence and poor survival [3–5]. CXCR4 enables migration towards its ligand CXCL12 (stromal cell-derived factor-1; SDF-1) present in common sites of metastasis such as the liver, lungs, lymph nodes and bone [6–8]. In addition to assisting in the dissemination of CRC, CXCR4 has also been shown to support growth of metastases through its co-expression with glycoprotein CD133, a marker of cancer stem cells [9].

It is now recognized that most tumors arise from a minority of cells capable of tumorigenesis [10]. These ‘cancer stem cells’ or ‘cancer initiating cells’ (CICs) possess the capacity to both self-renew and differentiate to re-populate the full phenotypic heterogeneity of the tumor [11]. Surface antigens used to identify CICs within CRC include CD133 [12] and the cell adhesion molecule CD44 [11], however their roles in metastatic disease remain controversial [13]. With the identification of CICs, the possibility that selective action of drugs may not eliminate the cell populations responsible for recurrence has received attention. Recently, a subpopulation of uniquely metastatic CICs has been reported to co-express CD26 [14].

CD26 is a multifunctional cell-surface protein that is variably expressed between different cancers but plays a role in regulating cancer progression and spread [15]. Its overexpression is linked to reduced invasiveness of ovarian cancer [16] and it is down-regulated during carcinogenesis of melanoma [17]. However, CD26 expression is up-regulated in renal cell carcinoma [18] as well as papillary and follicular thyroid carcinomas [19, 20]. High levels of CD26 expression is associated with worse survival in CRC [21].

That the consequence of CD26 expression by tumors remains equivocal is likely due to its diverse roles. In addition to directly facilitating adhesion to the extracellular matrix [22, 23], CD26 has two associated enzymatic activities: an intrinsic dipeptidyl peptidase IV (DPPIV; EC 3.4.14.5) activity and a hydrolase activity due to anchored ecto-adenosine deaminase (ADA; EC 3.5.4.4) for which CD26 is the major binding protein [15]. Clearance of adenosine by ADA can enhance immune surveillance, a process that is inhibited by the accumulation of adenosine in the tumor [24–26]. Through its peptidase activity, CD26 controls CXCL12 concentrations [27] and subsequent homing of hematopoietic progenitor cells to the bone marrow [28] along with the metastatic behavior of endometrial carcinoma [29] and Sézary cutaneous T cell lymphoma [30]. The contrasting effects of CD26 in different tumors may arise in part from the consequences of different levels of DPPIV activity, leading to a biphasic effect on the CXCL12 axis. Regional degradation of CXCL12 by DPPIV activity may serve to hone the CXCL12 gradient in a similar fashion to its second receptor CXCR7, preventing ligand-mediated receptor desensitization of CXCR4 [31], but higher levels of CD26 expression may result in gradient ablation.

Given the important roles of CXCR4 and CD26 in the tumorigenesis and metastatic outgrowth of CRC, we sought to observe whether their expression and function might change following exposure to anticancer agents. Our data show that the migratory phenotype of CRC cells is suppressed immediately following exposure to chemotherapeutic agents due to loss of CXCR4^+^ cells and elevation of CD26 peptidase; and this is associated with enrichment of a CD44^+^/CD133^−^ cell subset.

## Methods

### Cell culture

HT-29, T84, HRT-18, SW480 and SW620 human CRC cells and the additional cell lines mentioned were obtained from the American Type Culture Collection. Cells were cultured in Dulbecco’s modified Eagle’s medium (DMEM, without antibiotics) supplemented with 5 % (HT-29, T84, and HRT-18) or 10 % (SW480 and SW620) ^v^/_v_ heat-inactivated newborn calf serum (NCS) and maintained as stocks in 75-cm^2^ flasks at 37 °C in a humidified atmosphere of 90 % air/10 % CO_2_. Cells for use in binding assays or for measurements of DPPIV enzyme activity were seeded into 48-well plates at 50,000 cells/well and allowed to adapt to culture for 48 h. The cells were then cultured in medium containing 1 % NCS for a further 48 h. For flow cytometry, cells were seeded into 6-well plates and allowed to adapt for 48 h prior to treatment. Cells for migration assays were cultured and treated in 10-cm dishes. Anticancer agents used were: 5-fluorouracil (5-FU), irinotecan (IT), cisplatin (Cis), vinblastine (Vin), and methotrexate (MTX) from Mayne Pharma Canada; oxaliplatin (Ox) from Sanofi Canada; and SN-38 from Toronto Research Chemicals. Where single drug concentrations are used, these were defined as optimal (typically, just maximal) based upon the response of the cells at that passage level and the lot(s) of drug as obtained from the supplier(s).

### Assay for cell-surface CD26 and CXCR4 on cell monolayers

Cellular CD26 or CXCR4 protein levels were determined on cell monolayers at 4 °C as previously described [32]. Our assay for CD26 and CXCR4 measures native protein expressed at the surface of viable cells, rather than total cellular protein as with e,g, a western blot. Briefly, plates were placed on ice, and the cultures were washed with binding assay buffer (BAB; 137 mM NaCl, 5 mM KCl, 24.8 mM Tris, 0.7 mM Na_2_PO_4_, 0.5 mM MgSO_4_, 1 mM CaCl_2_, pH 7.4), containing 0.2 % ^w^/_v_ bovine serum albumin (BSA) followed by a 60-minute incubation with 125 μL BAB containing 1 % BSA and 1 μg/mL antibody or isotype-matched control. Primary mouse anti-human monoclonal antibody against CXCR4 (clone 12G5) and mouse IgG_2a_ isotype-matched control antibody (clone G155-178) were from BD Pharmingen, mouse anti-human monoclonal antibody against CD26 (clone M-A261) and mouse IgG1 (clone W3/25) isotype controls were from Cedarlane Laboratories, and secondary ^125^I-labeled goat anti-mouse IgG, F(ab’)_2_ fragment was obtained from PerkinElmer Life Sciences. After two washes with BAB containing 0.2 % BSA, the cells were incubated for 60 minutes with 125 μL BAB containing 1 % BSA and 1 μCi/mL ^125^I-labeled goat anti-mouse IgG, F(ab’)_2_ fragment. After three more washes the cells were solubilized in 0.5 M NaOH and radioactivity was counted. To determine antigen-specific radioactivity, the nonspecific binding in the presence of the isotype control antibody was subtracted from that obtained with the target antibody. Cell counts were performed with a Coulter® model ZM30383 particle counter (Beckman Coulter). Data are expressed as antigen-specific radioactivity (cpm) per 100,000 viable cells.

### Orthotopic tumor model

HT-29 cells (5 × 10^6^ in 100 μL serum-free DMEM) were injected s.c. into the flanks of six week old female CD-1 *nu/nu* mice (Charles River) and tumors were allowed to grow for 18–20 d until approximately 7 mm in diameter. The tumor tissue donors were euthanized under ketamine/xylazine anesthesia, tumors were harvested aseptically, and all non-tumor tissue was dissected away. The tissues were washed in ice-cold DMEM and cut into ~1 mm^3^ pieces for tumor transplantation. Recipient immunodeficient mice were anesthetized with 70 mg/kg ketamine and 14 mg/kg xylazine i.p. and treated proactively with 0.3 mg/kg buprenorphrine i.p. for post-surgical analgesia. A 1-cm abdominal incision was made to the right of midline and the distal small intestine was exteriorized to locate the ileocecal junction. The proximal end of the ascending colon was identified and abraded gently with the wooden end of a cotton-tipped applicator. Three 1-mm^3^ tissue pieces were sutured onto the muscularis of the proximal ascending colon, taking care not to pierce the colon wall. The intestine was interiorized and the incision was sutured. Twenty-six and 28 days following surgery, mice were weighed and injected i.p. with drugs or vehicle control (saline). Two days after the second dose, they were euthanized. The treatment and analysis period of days 26–30 represented the best time window between formation of an anatomically well-integrated tumour (by day 24) and a risk of occlusion of the intestinal lumen by the expanding tumour (from day 32) in the case of HT-29 cells. Tumors were harvested and tissues were weighed and snap-frozen in liquid nitrogen or fixed in 4 % formaldehyde for later analysis. All procedures were approved by the Carleton Animal Care Facility University Committee on Laboratory Animals at Dalhousie University.

### Immunolocalization of CD26 and CXCR4 in tumours

For visualisation of CD26, tumors were frozen in OCT® and sectioned at a thickness of 8 μm with a Leica CM 3050S cryostat (Leica Microsystems). Sections were mounted on slides and maintained at −20 °C. For immunohistochemistry, all steps were carried out at 4 °C, unless otherwise described. Sections were thawed briefly, rinsed with phosphate-buffered saline (PBS) containing 1 mg/mL BSA and 0.1 % Tween 20 (PBS/BSA/Tween), blocked with 3 % goat serum in PBS/BSA/Tween for 30 min, then incubated with 25 μL of PBS/BSA/Tween containing 5 μg/mL mouse anti-human CD26 primary antibody for 2 h in a humidified chamber. Sections were washed three times with PBS/BSA/Tween, and then incubated with 25 μL of PBS/BSA/Tween containing 2 μg/mL of an Alexa Fluor® 488-conjugated goat anti-mouse IgG secondary antibody for 2 h in a humidified chamber in the dark. Slides were washed a further three times, post-fixed with PBS containing 10 % formaldehyde for 10 min at room temperature, and rinsed with distilled water. Coverslips were mounted on sections using low-fade Gel/mount® and fluorescence was observed using a Leica DM 2000 fluorescence microscope (Leica Microsystems).

To observe CXCR4, formalin-fixed and paraffin wax-embedded tissue was sectioned and processed for immunoperoxidase procedures. Deparaffinised sections were subjected to antigen retrieval using 10 mM citrate buffer, pH 6.0 at 95 °C in a microwave. Rinsed sections were then stained for CXCR4 using the same procedure as for CD26, except that the bound primary antibody was identified using a Vectastain ABC kit (Vector laboratories, Burlingame, CA). Quantitation was performed in the absence of counterstain; the distribution of CXCR4 was visualised with a Harris’ hematoxylin counterstain.

Levels of CD26 or CXCR4 were analyzed with QCapture Pro® software. The average staining intensity of the tumor was measured in a randomly-selected area of constant dimension, determined by a blinded observer.

### Flow cytometry

HT-29 cells were released from 6-well plates by TrypLE™ Express. Cells were washed with chilled flow buffer (PBS, 25 mM HEPES, 1 mM EDTA, 1 % BSA) and resuspended in 2 μg/mL CXCR4-APC (clone 12G5; BD Pharmingen) and CD26-FITC (clone M-A261; Serotec), combined CD26-FITC (clone M-A261; Serotec), CD44-APC (clone G44-26; BD Pharmingen), CD133-PE (clone AC133; Miltenyi Biotec), or fluorophore-tagged isotype controls (Miltenyi Biotec) for 45 min at 4 °C. Cells were then washed twice with flow buffer and resuspended in BSA-free flow buffer for analysis. Flow cytometry analysis was carried out with a BD FACSCalibur™ flow cytometer (BD Biosciences). Cell debris and aggregates were excluded based on scatter signals and 10,000 events were captured per sample. Data were analyzed using Flowing Software version 2.5.0 (University of Turku, Turku, Finland).

Following labeling with CD26-PE (clone M-A261; Serotec) cells were stained with annexin-V-FITC and propidium iodide (PI) according to manufacturer’s protocol (Roche Diagnostics) for detection of necrotic, apoptotic, and live cells. Analysis was carried using a Guava® easyCyte™ 8HT flow cytometer and associated InCyte software (Millipore).

### DPPIV activity and ADA-binding capacity assays

DPPIV enzyme activity was measured spectrophotometrically using Gly-L-Pro *p*-nitroanilide (Gly-Pro-*p*NA; Sigma-Aldrich) as the DPPIV substrate [32]. To measure the cellular capacity for ecto-ADA binding, HT-29 cells in 48-well plates were treated with 10 μg/mL calf spleen ADA1 (Worthington Biochemical) in medium for 60 min at 37 °C and then assayed for bound ADA using 1 μg/mL rabbit anti-bovine ADA antibody (Alpha Diagnostic International) and 0.5 μCi/mL ^125^I-labeled donkey anti-rabbit secondary antibody F(abʹ)_2_ fragment (Amersham Biosciences), using the procedures previously described [32].

### Migration assays

Transwell® 8 μm pore size polycarbonate membrane inserts (Corning) were coated overnight at 37 °C with 1 μg/mL type V collagen. Drug- and vehicle-treated cells were released from culture by brief exposure to trypsin and resuspended at 0.5 - 2.5 × 10^6^ cells/mL in serum-free DMEM containing 1 mg/ml BSA. One hundred microlitres of cell suspension were added to the upper chamber, and 600 μL of DMEM containing 1 mg/mL BSA and 100 ng/mL CXCL12 or vehicle control were added to the bottom chamber. Chambers were incubated for 18 h at 37 °C, and filters were fixed and stained with Mayer’s hematoxylin. Cells remaining on the upper surface of the membrane were removed using a cotton-tipped applicator, and the filter was mounted using Cytoseal 60®. Cells that had migrated to the lower surface of the membrane were counted microscopically by a blinded independent observer.

### Statistical methods

Statistical significance of differences between data was determined using ANOVA or t-test models as indicated. Linear regression analysis was performed to identify parameters affecting the percent change in cells within a population after being subjected to a specific drug. Regression analysis and Tukey’s post hoc tests were completed using R version 3.0.2 (Vienna, Austria).

## Results

### Exposure to chemotherapeutic drugs leads to opposing changes in cell-surface expression of CXCR4 and CD26 in surviving populations of colon cancer cells

We first screened a range of human CRC cell lines (HT-29; adenocarcinoma, T84; CRC lung metastasis, HRT-18; ileocecal adenocarcinoma, SW480; primary adenocarcinoma, and SW620; secondary adenocarcinoma lymph node metastasis) for their endogenous expression of CXCR4 and CD26 using a radioimmunobinding assay, and evaluated possible changes in each marker in response to exposure to chemotherapeutic agents. Basal expression of CXCR4 varied between CRC cell lines with HT-29 cells having the highest expression (Table 1). Basal cellular expression of CD26 also varied greatly, with approximately 50-fold difference between cell lines and was broadly related to the degree of cellular differentiation, being highest in cells with a clear epithelial morphology (T84, HT-29), intermediate in cells that grow with a more extended morphology (HRT-18) and lowest in cells with rounded morphology and low substratum adherence (SW480, SW620) (Table 2). CD26 was present at approximately half the level at the surface of a metastasis-derived cell line SW620 compared with the paired cell line from the primary tumour SW480 [33] (Table 2).

**Table 1:** Constitutive expression and decrease of CXCR4 at the surface of human colorectal carcinoma cells due to anticancer drugs

Cell line	Basal CXCR4 expression (cpm/10^5^ cells)	Drug treatment			
		5-FU	Cis	Vin	MTX
		*Maximal decrease (% from control)*			
HT-29	470 ± 52	75 ± 6.1*	72 ± 6.3*	75 ± 12*	56 ± 7.6*
T84	220 ± 53	42 ± 15	48 ± 22	27 ± 4.4	39 ± 21
HRT-18	62 ± 27	59 ± 18	29 ± 11	76 ± 8.4	63 ± 27
SW480	230 ± 41	24 ± 13	32 ± 13	71 ± 7.6*	24 ± 6.4
SW620	80 ± 29	70 ± 7.0	77 ± 13*	72 ± 18	73 ± 3.0

The relative basal cell-surface protein expression for each cell line was summarized from 8–28 independent experiments. Cells were treated with drugs (5-FU, 5-fluorouracil [0.1 – 1,000 μg/mL]; Cis, cisplatin [0.001 – 10 μg/mL]; Vin, vinblastine [0.001 – 10 μg/mL]; MTX, methotrexate [0.1 – 1,000 μg/mL]) and cell surface expression of CXCR4 was determined by radioimmunobinding assay 48 h later. Maximal decrease of CXCR4 was recorded from 3–8 independent dose–response experiments for each drug and cell line. Data are shown as mean % decreases ± SE. *, significant % decrease due to drug, *P* < 0.05 using paired t-test

**Table 2:** Constitutive expression and increase of CD26 at the surface of human colorectal carcinoma cells by anticancer drugs

Cell line	Basal CD26 expression (cpm/10^5^ cells)	Drug treatment			
		5-FU	Cis	Vin	MTX
		*Maximal increase (% over control)*			
HT-29	1,300 ± 140	30 ± 5.8**	72 ± 26***	41 ± 10**	26 ± 6.4**
T84	780 ± 69	84 ± 14*	61 ± 42	78 ± 6.6	73 ± 42
HRT-18	370 ± 64	17 ± 9.1*	19 ± 9.1	23 ± 12	18 ± 9.4**
SW480	55 ± 15	96 ± 58*	90 ± 66	100 ± 59**	170 ± 150
SW620	26 ± 12	36 ± 31	28 ± 30	45 ± 43	53 ± 49

The relative basal cell-surface protein expression for each cell line was summarized from 12–5 independent experiments. Cells were treated with drugs (5-FU, 5-fluorouracil [0.1 – 1,000 μg/mL]; Cis, cisplatin [0.001 – 10 μg/mL]; Vin, vinblastine [0.001 – 10 μg/mL]; MTX, methotrexate [0.1 – 1,000 μg/mL]) and cell surface expression of CD26 was determined by radioimmunobinding assay 48 h later. Maximal up-regulation of CD26 was recorded from 3–7 independent dose–response experiments for each drug and cell line. Data are shown as mean % increases ± SE for the moderate/high CD26-expressing cell lines, and mean numerical increases (cpm/10^5^ cells) ± SE for the low expressers. *, significant increase due to drug, *P* < 0.05; **, *P* < 0.01; ***, *P* < 0.001, using paired t-test

These different cell lines were initially treated with chemotherapeutic agents that have entirely distinct mechanisms of action, so as to identify changes that would not be selective to any particular cellular target: 5-FU (pyrimidine antagonist), Cis (DNA cross-linker), Vin (microtubule poison), or MTX (folate antagonist). Following initial time course experiments (binding assays completed 0 – 72 h post-treatment; data not shown) cell-surface expression of CXCR4 and CD26 was determined at 48 h for subsequent experiments (Tables 1 and 2). Without exception and with the same effect or trend across five distinct CRC cell lines, each of these diverse agents led to a decrease in cell surface CXCR4 (Table 1) and conversely caused an increase in the net cell surface CD26 (Table 2).

In each case the maximal degree of change differed considerably depending upon the cell line and the responsiveness to perturbation was not the same for CXCR4 and CD26 (Tables 1, 2). For example, in the paired cell lines SW480 and SW620, which are derived from the same patient at different stages of CRC [33], SW480 cells (from the primary tumour) showed a more robust elevation of CD26 while SW620 cells (from a later metastasis) showed the more dramatic change in CXCR4. These and other observations (see below) suggested that regulation of changes in these two proteins were affected through different mechanisms.

### Decline in surface expression of CXCR4 and increase in CD26 is due to a drug-induced decrease in CXCR4+ cells and enrichment of CD26 expression in distinct populations

Due to significant basal levels of both CXCR4 and CD26 while demonstrating the most consistent changes to a range of different chemotherapeutics, we focused on the responses of HT-29 cells as our model system.

**Fig. 1:**
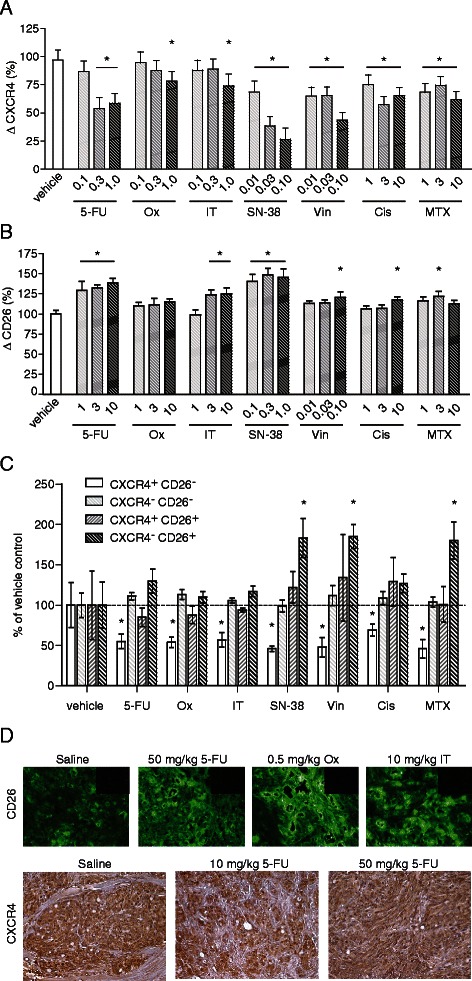
Anticancer agents decrease CXCR4 while increasing CD26 expression in the HT-29 cell line. HT-29 cells were treated with chemotherapeutic drugs (μg/mL) and stained for flow cytometry analysis of CXCR4 (**a**; mean % change in CXCR4 expression from vehicle control ± SE, *n* = 3; *, *P* < 0.01 by two-way ANOVA) or CD26 (**b**; mean % change in CD26 expression from vehicle control ± SE, *n* = 6; *, *P* < 0.05 by two-way ANOVA). **c** Dual-stain of CXCR4 and CD26 following drug treatment (5-FU = 1, Ox = 1, IT = 1, SN-38 = 0.1, Vin = 0.1, Cis = 1, MTX = 1 μg/mL; mean % of each population following vehicle control, *n* = 5; *, *P* < 0.01 by two-way ANOVA). **d** Mice (*nu*/*nu*) with HT-29 colorectal tumors growing within the large intestine were treated with saline, 10 or 50 mg/kg 5-FU, 0.5 mg/kg Ox, or 10 mg/kg IT four and two days before the tumors were harvested. Tumor sections were immunostained using anti-CD26 antibody or isotype control antibody (inserts); or are shown with peroxidase-stained CXCR4 with a hematoxylin counterstain. Representative images of four mice per group

We treated the cell population with chemotherapeutics including those used above but adding oxaliplatin (Ox; DNA cross-linker) and irinotecan (IT; topoisomerase 1 inhibitor), both drugs routinely used for treatment of human CRC [34]. We also evaluated the effect of the active metabolite of IT, SN-38. Treatment with the active agents progressively decreased the proportion of CXCR4^+^ cells in the cell population. The loss of CXCR4^+^ cells due to drug exposure showed substantial dose-dependent loss of CXCR4+ cells following treatment with agents 5-FU, Ox, Vin, Cis, MTX and SN-38 at the concentrations tested (Fig. 1a, Additional file 1: Figure S1a).

Treatment with 5-FU, Vin, Cis, and MTX consistently elevated CD26 (Fig. 1b, Additional file 1: Figure S1b), reflective of a general up-regulation of CD26. Both SN-38 and IT were capable of elevating CD26 when used at higher concentrations than Fig. 1a, although SN-38 was more potent as expected (Fig. 1b).

Co-staining of both CXCR4 and CD26 revealed roughly equal representation of CXCR4^+^/CD26^−^, CXCR4^−^/CD26^+^, and CXCR4^+^/CD26^+^ populations, with a majority (~70 %) of cells staining CXCR4^−^/CD26^−^. At the moderate concentrations used, all drug treatments substantially reduced the proportion of CXCR4^+^/CD26^−^ cells, with little change in proportions of CXCR4^−^/CD26^−^ and CXCR4^+^/CD26^+^ populations (Fig. 1c). As expected, SN-38, Vin, MTX, and to a lesser extent 5-FU enriched for CXCR4^−^/CD26^+^ cells (Fig. 1c).

### Increase in CD26 and decline in tumour cell expression of CXCR4 occurs in vivo and is not an artefact of cytotoxicity

We sought to confirm our results using an orthotopic mouse model of *nu*/*nu* mice with HT-29 colorectal tumors growing within the large intestine. Established tumors were treated systemically with saline or 5-FU, Ox or IT prior to being stained by immunofluorescence (CD26) or immunoperoxidase (CXCR4) (Fig. 1d). Tumors of treated mice showed a marked increase in CD26 protein based on fluorescent intensity compared to controls (*P* < 0.05), consistent with *in vitro* data from cell monolayers. Interestingly Ox, which showed little potency to elevate CD26 *in vitro*, was the most effective in enhancing CD26 *in vivo* (Fig. 1d; mean increase 57 % compared with 28 % for 5-FU and 48 % for IT), showing that additional influences may modify the phenomenon in the whole animal.

A decline in CXCR4 within the tumour parenchyma due to drugs was also observed after staining with immunoperoxidase (Fig. 1d), although quantitative analysis showed statistical significance only with the 50 mg/kg dose of 5-FU (45.7 ± 1.5 compared with 55.9 ± 3.5 for the saline control, *P* = 0.01). Quantification of tumour cell CXCR4 was complicated by tumour heterogeneity and the expression of CXCR4 on other cells within the tumour tissue (compare controls in Fig. 1d).

Given the acute treatment regimen and follow-up period, no effect on tumor growth was generally observed at time of sacrifice compared to saline-treated mice, with the exception of the mice treated with the highest dose (50 mg/kg) of 5-FU, which exhibited a statistically significant weight loss (4 %; 1.0 ± 0.4 g).

Although flow cytometry events were gated to eliminate nonviable cells, we sought to exclude the possibility that changes in these surface proteins at high drug doses might be a consequence of toxicity. Staining with annexin V-PI was used to distinguish between viable cells and cells in the early stages of apoptosis or necrosis. Data for CD26, which responded at higher drug doses, are shown in Additional file 2: Figure S2 and reveal that only live (annexin V^−^/PI^−^) cells contributed to increased CD26 levels following drug treatment. To determine whether changes in overall expression were confined to the cell surface or due to changes in gene expression, the fold-change of CXCR4 and CD26 mRNA following 12 hour treatment was quantified by qPCR (Additional file 3: Figure S3, Additional file 5: Supplementary Methods).

### Chemotherapeutic drugs consistently decrease CXCR4 and elevate CD26 protein expression on human CRC cells but the two responses have different kinetics and maxima

Time courses of changes in cell-surface proteins in response to drug treatments differ between CXCR4 and CD26, but in general begin 24–72 h after drug addition (data not shown). A more comprehensive assessment of the decrease in CXCR4^+^ cells with a range of chemotherapeutic agents (Additional file 5: Table S1) showed that the maximum decrease averaged 78.6 % and was not significantly different between six drug treatments (*P* = 0.26), consistent with the view that different agents act by eliminating the fixed proportion of CXCR4+ cells. The maximum increase in CD26 expression, however, varied greatly as a consequence of different drug treatments (Additional file 5: Table S1), ranging from 22 % for Ox to 72 % for Cis. This again is consistent with the expectation that there is an elevation of CD26 on all surviving cells, which will be dependent upon signalling pathways that are initiated differentially by different drugs. The two extremes of maximal effect for an increase in CD26 exist for two agents (oxaliplatin and cisplatin) that have the same mechanism of cytotoxicity; suggesting that the mode of up-regulation of CD26 is separable from pathways that lead to cell death.

### Increased CD26 cell-surface expression is accompanied by elevated DPPIV activity and ecto-ADA binding following chemotherapeutic drug treatment

**Fig. 2:**
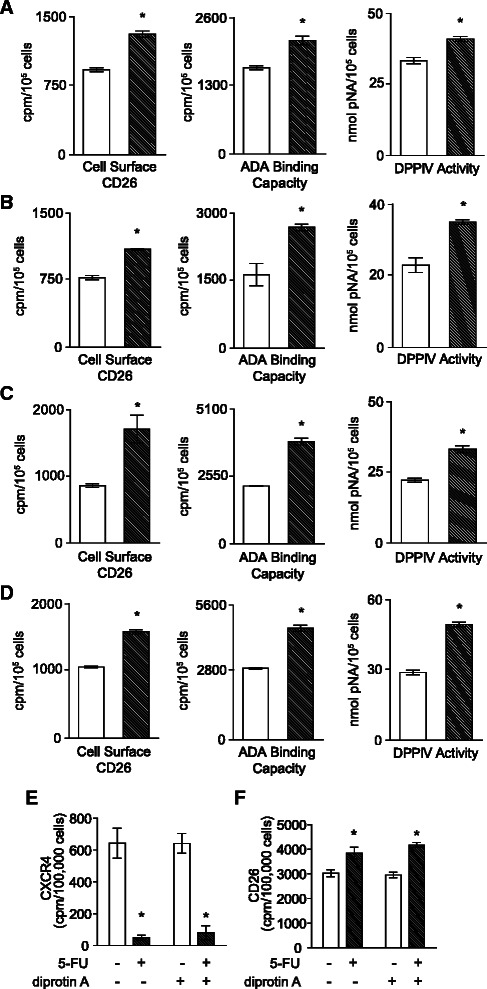
Adenosine deaminase-binding capacity and dipeptidyl peptidase IV activity increase in parallel with CD26 protein. Cells were treated with vehicle (light bars) or 2 μg/mL **a** 5-FU; **b** Cis; **c** Vin; or **d** MTX (hatched bars) and assayed when the maximal effect was attained, at 24 h (Vin) or 48 h (5-FU, Cis, MTX) for CD26 protein, ADA-binding capacity, and DPPIV activity. Mean ± SE (*n* = 4). *, *P* < 0.01 by Student’s t-test. **e** Decrease in CXCR4 due to 20 μg/mL 5-FU is maintained in the presence of the DPPIV enzyme inhibitor diprotin A (75 μM). **f** Increase in CD26 due to 20 μg/mL 5-FU is maintained in the presence of the DPPIV enzyme inhibitor diprotin A. Mean ± SE (*n* = 4). *, *P* < 0.01 by ANOVA

CD26 is the major cellular anchoring protein for ecto-ADA and has an intrinsic dipeptidyl peptidase activity [15], therefore we examined the functional consequence of increased CD26 on HT-29 cells directly in parallel with measurements of the surface abundance of CD26 (Fig. 2). Treatment with 5-FU (Fig. 2a), Cis (Fig. 2b), Vin (Fig. 2c), or MTX (Fig. 2d) revealed closely comparable proportional (%) increases in ecto-ADA binding capacity and DPPIV activity compared with those for CD26 protein, showing that the additional CD26 at the cell surface is functional.

We have shown that ecto-ADA plays a role in the auto-regulation of CD26/DPPIV in that it permits increased down-regulation of CD26/DPPIV due to adenosine in the hypoxic tumour environment [32]. Moreover, decreases in DPPIV activity permit increased levels of CXCL12, which is the major substrate of DPPIV [35]. Increased CXCL12 may then down-regulate CXCR4 [36]. We therefore questioned whether changes in these enzyme activities could be responsible for the concomitant regulation of CXCR4 with CD26. However, an increase in ecto-ADA would reduce ambient adenosine, which our earlier findings indicate would not reduce CXCR4 expression [37]. Inhibition of DPPIV with diprotin A did not alter 5-FU- induced down-regulation of CXCR4 (Fig. 2e). Similarly, diprotin A inhibition of DPPIV did not alter 5-FU-induced CD26 up-regulation (Fig. 2f). We conclude that secondary alterations in ecto-ADA and DPPIV activities do not contribute to the linked and opposing changes in CXCR4 and CD26 after chemotherapeutic drug exposure.

### Exposure of CRC cells to chemotherapeutic drugs eliminates subsequent CXCL12-driven cell migration

**Fig. 3:**
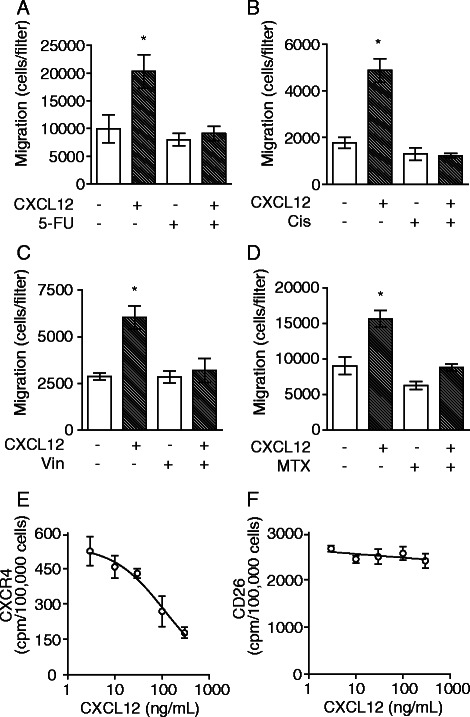
Prior treatment with anticancer agents significantly diminishes chemotaxis toward CXCL12. Cells were pre-treated either with vehicle control or 2 μg/mL **a** 5-FU; **b** Cis; **c** Vin; or **d** MTX for 48 h, isolated from culture and then assayed for chemotaxis toward 100 ng/mL CXCL12 (hatched bars) or vehicle control (light bars). Mean ± SE (*n* = 6). *, *P* < 0.01 by ANOVA. **e** CXCL12-dependent loss of CXCR4 cell-surface expression. **f** cell-surface CD26 is unaffected by CXCL12

Metastasis and growth of many cancers is driven by CXCL12 through CXCR4 and may be modulated by DPPIV-mediated CXCL12 degradation [29, 38]. HT-29 cells that had been exposed to drugs were tested for their migration toward CXCL12 in a chemotaxis assay (Fig. 3a-d). The surviving cells remained motile following treatment with 5-FU (Fig. 3a), Cis (Fig. 3b), Vin (Fig. 3c), or MTX (Fig. 3d), showing the same background ability to cross the collagen-coated polycarbonate filters as untreated cells over an 18 h period. However, pre-treatment with any of these agents completely abrogated CXCL12-mediated migration, showing that the reduction in CXCR4 when accompanied by enhancement of functional CD26 leads to a complete suppression of migration toward CXCL12, and extends observations of reduced migration of SW480 towards CXCL12 in the presence of irinotecan [39]. Treatment with CXCL12 for 48 hours resulted in decreased cell-surface CXCR4 as expected (Fig. 3e), without altering CD26 levels (Fig. 3f).

### Anticancer agents enrich for CD26+ and CD44+ populations while decreasing CD133- populations

Pang and colleagues [14] have provided evidence that CD26 is a marker of metastatic CRC stem cells. However, the latter finding conflicts with the long-established belief that CD26, as the major intestinal ADA-binding protein and DPPIV [15], is a marker of differentiated epithelial cells in the intestine [40, 41]. For this reason we examined the co-expression of established CRC ‘stem cell’ (CIC) markers CD44 and CD133 alongside CD26.

**Fig. 4:**
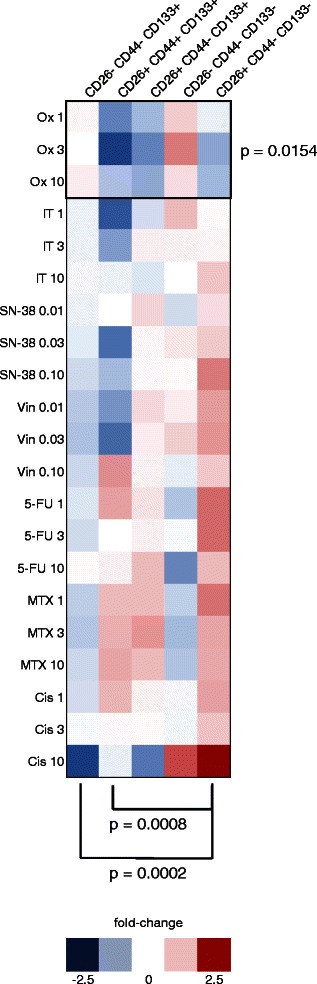
Percent change of cell populations following treatment with anticancer agents. Heatmaps of fold-change in the five CD26/CD44/CD133 populations present within HT-29 cultures at a frequency >1 % (*n* = 6). Oxaliplatin (Ox) was identified as a covariate having a significantly different effect on cell populations. Brackets highlight significant differences between percent changes in populations following drug treatment

Triple staining of HT-29 cells for CD26, CD44 and CD133 revealed consistent changes in response to treatment with chemotherapeutic drugs. Regression results from a model fitting to the percent change in number of cells as a function of principal cell populations (frequency >1 %) and drugs (Marker and Drug variables) are shown in Additional file 5: Table S2 and the results of *post hoc* tests for percent change between cell populations are shown in Additional file 5: Table S3. Residual checks showed no departure from the assumptions of normality, independence and constant variance. Individual significance tests of the regression coefficients show that ‘DrugOx’ (oxaliplatin) had a significant effect on the average percent change in the number of cells with respect to a control group, while controlling for the marker populations. Populations CD26^−^/CD44^−^/CD133^+^ and CD26^+^/CD44^−^/CD133^−^ also had a statistically significant contribution to the response (Additional file 5: Table S2). Tukey’s *post hoc* tests for percent change between cell populations revealed CD26^+^/CD44^−^/CD133^−^ cells differed significantly from CD26^−^/CD44^−^/CD133^+^ and CD26^+^/CD44^+^/CD133^+^ populations in their response to anticancer agents (Fig. 4, Additional file 5: Table S3). The changes across drug treatments within the five major populations and the statistical differences are summarised in Fig. 4. Populations CD26-/CD44+/CD133+, CD26-/CD44+/CD133-, and CD26 + CD44 + CD133- each represented less than one percent of all cells.

Within subpopulations, cells treated with drugs having substantially diverse mechanisms of action showed general decline in CD133 (Additional file 4: Figure S4a) and increases in CD44 (Additional file 4: Figure S4b) and CD26 (Additional file 4: Figure S4c). Increases in CD26 expression were seen across all combinations of cellular expression of CD133 and CD44. However overall CD26 elevation was most marked in association with the CD44^+^/CD133^−^ phenotype and least associated overall with the opposite CD44^−^/CD133^+^ phenotype (Additional file 4: Figure S4c). Interestingly, Ox-treated cells were distinctive in that CD26 expression actually declined in association with CD44^−^/CD133^+^ cells in contrast to all other drug treatments (Additional file 4: Figure S4c).

## Discussion

Drivers in the recurrence of CRC include the chemokine receptor CXCR4, its ligand CXCL12, and the ectoenzyme CD26 for which CXCL12 is a substrate [35]. We find that this regulatory framework is altered in the population of surviving cells following incomplete eradication by chemotherapeutic agents.

A diverse range of established cytotoxic agents share the ability to down-regulate CXCR4 but up-regulate CD26, in this case on five different CRC cell lines (T84, HT-29, HRT-18, SW480, SW620). Furthermore, suppression of the CXCL12-CXCR4 axis due to loss of CXCR4 is potentially accentuated by this concomitant up-regulation of CD26, because its DPPIV activity degrades CXCL12 and there is therefore a parallel reduction in the bioactivity of the chemokine ligand as well as the decline in its receptor. These effects on CXCR4 and CD26 were independent of the cytotoxic mechanisms of action of these drugs, which acted through DNA cross-linking (Cis, Ox), inhibition of DNA synthesis (5-FU, MTX), microtubule disruption (Vin) or topoisomerase inhibition (IT; SN-38). The ability of agents (e.g. MTX, VB) that are not components of current cytotoxic regimens may allow them to be added in late-stage disease to manipulate CXCR4/CD26 levels without contributing to existing dose-limiting toxicities.

Loss of the average CXCR4 expression of the cell population was due to selective elimination of CXCR4^+^ cells. The same diverse collection of agents caused an increase in the abundance of CD26 at the CRC cell surface after treatment. In this case the change in CD26 did not result from selection toward a greater proportion of CD26^+^ cells, but an overall increase in CD26 net cell-surface expression. This was found to be (i) an authentic increase on viable cells, (ii) an increase in fully-functional CD26 (able to bind ecto-ADA and with DPPIV activity), (iii) independent of ambient levels of CXCL12, and (iv) regulated separately from DPPIV activity (as was the decrease in CXCR4). (This phenomenon of chemotherapy-induced CD26 elevation was also reproducible in four other, non-CRC carcinoma cell lines (LNCaP and PC-3; prostate, A549; lung, and T-47D; breast) and a non-epithelial cancer cell line (SH-SY5Y; neuroblastoma); data not shown).

The mechanism of chemotherapeutic drug-induced elevation of CD26 clearly differs from the simple selective process of CXCR4 and remains under investigation. For most drugs the EC_50_ for CD26 elevation did not differ significantly from that for reduction of CXCR4, although for cisplatin the concentration required to elevate CD26 was 10-fold higher than that required to reduce CXCR4^+^ cells, indicating that the mechanisms are not linked in a consistent manner. Furthermore, the maximum decrease in CXCR4 was essentially the same for all drugs at around ~ 80 % (Additional file 5: Table S1), consistent with a cell selection process in which the outcome is defined by the survival of CXCR4+ cells. However, the maximum achievable elevation for CD26 varied between 22 % and 72 %, revealing differences in how this outcome is signalled. These two extremes in fact corresponded to the two agents – Cis and Ox – that are the most similar in their cytotoxic mode of action of all the major drugs studied, suggesting a distinction from pathways that lead to cell death. It was also noted that IT was more able to elevate CD26 than would be expected if compared with its active metabolite SN-38. The data overall point to a substantially different pathway by which these agents up-regulate CD26, albeit one that is initiated by many cytotoxic agents. It is worth noting that general approaches to kill CRC cells by nonspecific means (exposure to pH 8.6, 300 mM NaCl or 0.01 % w/v deoxycholate) neither elevated CD26 nor selected for an altered CXCR4+ subpopulation (data not shown).

HT-29 cells that had survived treatment with chemotherapeutic agents at modestly cytotoxic concentrations were fully proficient in terms of their capacity for movement at 48 h after treatment - basal migration did not differ from that of untreated cells. However, the directed migration toward CXCL12 was completely ablated. We attribute this loss of migration towards CXCL12 to the decline in CXCR4 and concomitant increase in DPPIV activity through gain of CD26. Comparable treatment with these drugs had no effect on the (low) levels of the alternate CXCL12 receptor CXCR7 expressed at the surface of human CRC cell lines (data not shown).

Outgrowth of residual disease has been attributed to a subpopulation of drug-resistant CICs capable of differentiating into the different cellular hierarchies that make up the overall tumor bulk [11, 42]. Chronic treatment of CRC cell lines with 5-FU or Ox enriches for CIC markers CD44 and CD133 [43], and irinotecan-treated xenografts show a greater frequency of CD44^+^ cells [44]. This implicates both CD44 and CD133 as markers for the putative CRC CIC subset. Although classically recognized as a marker of differentiation [40, 45], CD26 expression has recently been reported to define a metastatic subpopulation of CICs within CRC and associated with development of metastasis in CRC patients [14, 21]. The paradox of CD26 being both a differentiation marker and correlate of cancer aggression likely reflects its multiple roles [15] and the consequence that its impact on cell function is context dependent.

Using primary colon cancer cells 5-FU has been reported to increase the CD26^+^/CD133^+^ subpopulation [14]. This is consistent with our finding that CD26 is increased across all cell subpopulations after 5-FU treatment. We do however extend the observation to show that many agents increase CD26 levels on CRC cells, and report for the first time that treatment with the agents currently used to treat CRC favors the emergence of a CD26^+^/CD44^+^ cell population. This is an intriguing finding in that there is increased co-expression of two markers both interact with the extracellular matrix and might be able to cooperate in the process of metastasis associated with CD26 identified by Pang and colleagues [14]. CD26 is known to associate with both fibronectin and collagen [46, 47] while CD44 is a receptor for hyaluronic acid (HA) and functions in cell adhesion and tumorigenicity [48, 49].

## Conclusion

Collectively, our data show that an important signaling mechanism of cancer metastasis may be down-regulated in parallel to the cytotoxic effects of chemotherapy. As well as adding to our knowledge of how existing agents work, this may have implications for understanding the cellular phenotype in residual disease after chemotherapy, particularly where a less dose intense approach is used. Understanding the post-treatment cellular phenotype, and why some patients relapse despite therapy is crucial. Phenotyping of circulating tumor cells post-chemotherapy offers a sensitive method of detecting surviving cancer cells following adjuvant therapy [50]. Our findings suggest that it will be productive to evaluate the CD26/CD44/CD133/CXCR4 status of refractory cancer cells in future studies of CRC.

## Additional files

Additional file 1: Figure S1. Anticancer agents decrease CXCR4^+^ cells and increase cell-surface expression of CD26 in the HT-29 cell line. **(A)** Representative histograms showing the loss of CXCR4^+^ cells following treatment with 5-FU or SN-38 as indicated, with concentrations in μg/mL. **(B)** Representative histograms showing the overall enhancement (rightward shift) following treatment with 5-FU or SN-38 as indicated (concentrations in μg/mL). Grey lines show frequency distributions of vehicle control-treated cells. (EPS 972 kb)
Additional file 2: Figure S2. Chemotherapeutic drugs increase CD26 protein expression on live cells but do not alter immunoreactivity against dead cells. HT-29 cells were co-stained with CD26-PE-annexin V-PI and gated to necrotic (**A**; annexin V^+^/PI^+^), apoptotic (**B**; annexin V^+^/PI^−^), or live (c; annexin V^−^/PI^−^) populations following treatment. Mean channel fluorescence of CD26-PE ± SE (*n* = 6). *, *P* < 0.01 compared to vehicle control by two-way ANOVA. (EPS 833 kb)
Additional file 3: Figure S3. Fold-change in CXCR4 and CD26 mRNA expression following treatment with anticancer agents. Cells were pre-treated either with vehicle control or 2 μg/mL 5-FU, Cis, Vin, or MTX for 12 h, isolated from culture and then assayed for CXCR4 (grey bars) or CD26 (white bars) mRNA qPCR by SYBR green. Mean ± SE (*n* = 3). (EPS 511 kb)
Additional file 4: Figure S4. Treatment with diverse anticancer agents increases CD26 while also enhancing CD44+, but lowering CD133+ populations. Heatmaps of fold-change in cellular expression of **(A)** CD133, **(B)** CD44, and **(C)** CD26 within different CRC subpopulations separated by pairs of the alternative markers (*n* = 6). (EPS 812 kb)
Additional file 5: **Supplementary Methods: Real-time (q)PCR.** **Table S1:** Comparison of the effects of chemotherapeutic agents on cell-surface CXCR4 and CD26 on remaining viable HT-29 cells following 48 h of exposure. **Table S2:** Estimation results for regression model with cell Marker and Drug as covariates. **Table S3:** Confidence level tests for percent change between cell populations. (DOC 53 kb)

## Abbreviations

5-FU: 5-fluorouracil
ADA: Ecto-adenosine deaminase
ANOVA: Analysis of variance
BAB: Binding assay buffer
BSA: Bovine serum albumin
CD133: Cluster of differentiation 133
CD26: Cluster of differentiation 26
CD44: Cluster of differentiation 44
CIC: Cancer initiating cell
Cis: Cisplatin
cpm: counts per minute; specific radioactivity
CRC: Colorectal cancer
CXCL12: C-X-C motif ligand 12
CXCR4: C-X-C motif receptor 4
Df: Degrees of freedom
DMEM: Dulbecco’s modified Eagle’s medium
DPPIV: Dipeptidyl peptidase IV
EC_50_: Half maximal effective concentration
EDTA: Ethylenediaminetetraacetic acid
FITC: Fluorescein isothiocyanate
GAPDH: Glyceraldehyde 3-phosphate dehydrogenase
HEPES: 4-(2-hydroxyethyl)-1-piperazineethanesulfonic acid
IT: Irinotecan
M-MLV: Maloney murine leukemia virus
MTX: Methotrexate
NCS: Newborn calf serum
Ox: Oxaliplatin
PBS: Phosphate-buffered saline
PCR: Polymerase chain reaction
PE: Phycoerythrin
PI: Propidium iodide
pNA: p-nitroanilide
qPCR: quantitative polymerase chain reaction
SDF-1: Stromal cell-derived factor-1
SE: Standard error
Vin: Vinblastine
