# Supplementary material for: Chemotherapeutic agents attenuate CXCL12-mediated migration of colon cancer cells by selecting for CXCR4-negative cells and increasing peptidase CD26
Source: BMC Cancer. 2015 Nov 10;15:882. doi: 10.1186/s12885-015-1702-2 (PMC4640216; doi:10.1186/s12885-015-1702-2)
Supplement: Additional file 5: — Supplementary Methods: Real-time (q)PCR. Table S1: Comparison of the effects of chemotherapeutic agents on cell-surface CXCR4 and CD26 on remaining viable HT-29 cells following 48 h of exposure. Table S2: Estimation results for regression model with cell Marker and Drug as covariates. Table S3: Confidence level tests for percent change between cell populations. (DOC 53 kb) [file 12885_2015_1702_MOESM5_ESM.doc]

**Supplementary methods**

**Real-time (q)PCR**

Brilliant SYBR® Green kits were from Stratagene (Cedar Creek). All other supplies for real-time PCR were from Invitrogen Canada. Total RNA was isolated using TRIzol® according to the manufacturer’s protocol, and 5 μg RNA were reverse-transcribed using M-MLV reverse transcriptase and oligo(dT)_12-18_ primer. Brilliant SYBR® Green was combined with cDNA and primers specific for CXCR4 (forward: 5’-gcctgagtgctccagtagcc-3’; reverse: 5’-tggagtcatagtcccctgagc-3’) or GAPDH (forward: 5’-catgagaagtatgacaacagcct-3’; reverse: 5’-agtccttccacgataccaaagt-3’), and real-time PCR amplification was performed using a Stratagene Mx3000P system (Cedar Creek). Relative CXCR4 expression was determined using the 2^-ΔΔCt^ method, with standardization against GAPDH and normalization to vehicle treatment.

**Supplementary Results**

**Table S1 Comparison of the effects of chemotherapeutic agents on cell-surface CXCR4 and CD26 on remaining viable HT-29 cells following 48 h of exposure.** HT-29 cells were treated as shown and CXCR4 and CD26 assayed 48 h later. Data are mean values ± SE for 3-11 independent experiments. One-way ANOVA with Bonferroni’s post-test. n.s., not significant.

Decrease in cell-surface Increase in cell-surface

CXCR4 on remaining cells CD26 on remaining cells

________ _____

Drug Maximum EC_50_ (μg/mL) Maximum EC_50_ (μg/mL) Difference

reduction (%) increase (%) in EC_50_

5-FU 81 ± 7.4 0.78 ± 0.25 30 ± 5.8 1.1 ± 0.30 n.s.

Cis 89 ± 9.5 1.6 ± 0.32 72 ± 26 17 ± 10 P < 0.05

Vin 87 ± 9.2 0.0044 ± 0.0014 42 ± 10 0.014 ± 0.007 n.s.

MTX 62 ± 6.3 6.4 ± 6.4 26 ± 6.4 0.44 ± 0.26 n.s.

Ox 70 ± 7.3 1.0 ± 0.16 22 ± 2.8 1.5 ± 1.5 n.s.

**Table S2 Estimation results for regression model with cell Marker**

**and Drug as covariates.**

| Covariates | Estimate | Std. Error | t value | Pr(>|t|) |
| --- | --- | --- | --- | --- |
| (Intercept) | 111.6716 | 7.1695 | 15.5759 | 0.0000 |
| MarkerCD26- CD44- CD133+ | -13.7583 | 6.8326 | -2.0136 | **0.0469** |
| MarkerCD26+ CD44- CD133- | 18.0529 | 6.9228 | 2.6077 | **0.0106** |
| MarkerCD26+ CD44- CD133+ | -1.6686 | 6.8326 | -0.2442 | 0.8076 |
| MarkerCD26+ CD44+ CD133+ | -10.9943 | 6.8326 | -1.6091 | 0.1110 |
| DrugCis | -6.2822 | 8.2335 | -0.7630 | 0.4474 |
| DrugIT | -12.8320 | 8.0844 | -1.5873 | 0.1158 |
| DrugMTX | 2.8344 | 8.0844 | 0.3506 | 0.7267 |
| DrugOx | -19.9623 | 8.0844 | -2.4692 | **0.0154** |
| DrugSN-38 | -8.6678 | 8.0844 | -1.0722 | 0.2864 |
| DrugVin | -5.7921 | 8.0844 | -0.7165 | 0.4755 |

|  | Df | Sum Sq | Mean Sq | F value | Pr(>F) |
| --- | --- | --- | --- | --- | --- |
| Marker | 4 | 12690.80 | 3172.70 | 6.47 | **1.2E-04** |
| Drug | 6 | 5280.40 | 880.07 | 1.80 | 0.11 |
| Residuals | 93 | 45586.84 | 490.18 |  |  |

Df, degrees of freedom

**Table S3** **Tukey’s 95% family wise confidence level tests for percent change**

**between cell populations** (order listed as CD26/CD44/CD133).

| Pairs | Difference | Lower | Upper | p-value |
| --- | --- | --- | --- | --- |
| --+ vs. --- | -13.7583 | -33.1961 | 5.6795 | 0.2899 |
| +-- vs. --- | 18.0049 | -1.6744 | 37.6842 | 0.0896 |
| +-+ vs. --- | -1.6686 | -21.1064 | 17.7692 | 0.9993 |
| +++ vs. --- | -10.9943 | -30.4321 | 8.4435 | 0.5190 |
| **+-- vs. --+** | **31.7632** | **12.0839** | **51.4425** | **0.0002** |
| +-+ vs. --+ | 12.0897 | -7.3481 | 31.5275 | 0.4216 |
| +++ vs. --+ | 2.7640 | -16.6738 | 22.2018 | 0.9948 |
| +-+ vs. +-- | -19.6735 | -39.3528 | 0.0058 | 0.0501 |
| **+++ vs. +--** | **-28.9992** | **-48.6785** | **-9.3199** | **0.0008** |
| +++ vs. +-+ | -9.3257 | -28.7635 | 10.1121 | 0.6713 |
